# Poly(ADP‐ribose)‐mediated interplay of XPA and PARP1 leads to reciprocal regulation of protein function

**DOI:** 10.1111/febs.12885

**Published:** 2014-07-21

**Authors:** Jan M. F. Fischer, Oliver Popp, Daniel Gebhard, Sebastian Veith, Arthur Fischbach, Sascha Beneke, Alfred Leitenstorfer, Jörg Bergemann, Martin Scheffner, Elisa Ferrando‐May, Aswin Mangerich, Alexander Bürkle

**Affiliations:** ^1^ Molecular Toxicology GroupDepartment of BiologyUniversity of KonstanzGermany; ^2^ Konstanz Research School Chemical BiologyUniversity of KonstanzGermany; ^3^ Department of Life SciencesAlbstadt‐Sigmaringen University of Applied SciencesSigmaringenGermany; ^4^ Research Training Group 1331University of KonstanzGermany; ^5^ Department of Physics and Center for Applied PhotonicsUniversity of KonstanzGermany; ^6^ Laboratory of Cellular BiochemistryDepartment of BiologyUniversity of KonstanzGermany; ^7^ Bioimaging CenterUniversity of KonstanzGermany; ^8^ Max Delbrück Center for Molecular MedicineBerlinGermany; ^9^ Institute of Pharmacology and ToxicologyUniversity of Zurich/VetsuisseZurichSwitzerland

**Keywords:** ARTD1, host cell reactivation assay, laser microirradiation, nucleotide excision repair, xeroderma pigmentosum

## Abstract

Poly(ADP‐ribose) (PAR) is a complex and reversible post‐translational modification that controls protein function and localization through covalent modification of, or noncovalent binding to target proteins. Previously, we and others characterized the noncovalent, high‐affinity binding of the key nucleotide excision repair (NER) protein XPA to PAR. In the present study, we address the functional relevance of this interaction. First, we confirm that pharmacological inhibition of cellular poly(ADP‐ribosyl)ation (PARylation) impairs NER efficacy. Second, we demonstrate that the XPA–PAR interaction is mediated by specific basic amino acids within a highly conserved PAR‐binding motif, which overlaps the DNA damage‐binding protein 2 (DDB2) and transcription factor II H (TFIIH) interaction domains of XPA. Third, biochemical studies reveal a mutual regulation of PARP1 and XPA functions showing that, on the one hand, the XPA–PAR interaction lowers the DNA binding affinity of XPA, whereas, on the other hand, XPA itself strongly stimulates PARP1 enzymatic activity. Fourth, microirradiation experiments in U2OS cells demonstrate that PARP inhibition alters the recruitment properties of XPA‐green fluorescent protein to sites of laser‐induced DNA damage. In conclusion, our results reveal that XPA and PARP1 regulate each other in a reciprocal and PAR‐dependent manner, potentially acting as a fine‐tuning mechanism for the spatio‐temporal regulation of the two factors during NER.

**Abbreviations**
6‐4PP: pyrimidine (6‐4) pyrimidone photoproduct
CPD: cyclobutane pyrimidine dimer
DDB2: DNA damage‐binding protein 2
eGFP: enhanced GFP
EMSA: electrophoretic mobility shift assay
ERCC1: excision repair cross‐complementing repair deficiency complementation group 1
GFP: green fluorescent protein
HRP: horseradish peroxidase
NER: nucleotide excision repair
PAR: poly(ADP‐ribose)
PARG: poly(ADP‐ribose) glycohydrolase
PBM: PAR‐binding motif
PFA: paraformaldehyde
TCA: trichloroacetic acid
TFIIH: transcription factor II H
XPA: xeroderma pigmentosum, complementation group A

## Introduction

Poly(ADP‐ribosyl)ation (PARylation) is a complex post‐translational modification observed in higher eukaryotes. Upon different stimuli, such as genotoxic and other forms of cellular stress, enzymes of the family of PARPs [poly(ADP‐ribose) polymerases], also known as ADP‐ribosyltransferases with diphtheria toxin homology; ARTDs), use NAD^+^ as a substrate to synthesize the biopolymer poly(ADP‐ribose) (PAR) with a variable chain length consisting of up to 200 ADP‐ribose moieties 1. Within the PARP family, PARP1 accounts for the bulk of cellular PARylation activity, in particular upon binding to DNA strand breaks under conditions of genotoxic stress 4. Importantly, high amounts of PAR are only transiently present in the cell because PAR is rapidly hydrolyzed by poly(ADP‐ribose) glycohydrolase (PARG) 5. The modification of target proteins occurs via two different modes: either PAR is covalently attached to specific amino acids, such as Glu, Asp and Lys, or proteins interact with PAR noncovalently via at least five different PAR‐binding modules 7. Thereby, PARylation acts as a highly dynamic post‐translational modification that controls the spatio‐temporal localization and function of its target proteins 1. Accordingly, PARylation is involved in multiple cellular processes such as DNA/RNA metabolism, proteostasis, chromatin modification, transcription, cell cycle regulation and cell death 9. In particular, PARP1‐dependent PARylation participates in the repair of DNA single and double‐strand breaks 3. Moreover, several reports imply a role of PARP1 in nucleotide excision repair (NER) 11. NER is the principal repair mechanism for the removal of bulky helix‐distorting DNA adducts, such as UV‐light‐induced cyclobutane pyrimidine dimers (CPDs) or pyrimidine (6‐4) pyrimidone photoproducts (6‐4PP) 18. Two distinct modes of NER are known: global genome repair and transcription coupled repair. Whereas, in transcription coupled repair, DNA damage signaling is mediated via CSA/CSB proteins, global genome repair relies on the damage recognition by XPC and the UV–DDB complex (DDB1‐DDB2‐containing E3‐ubiquitin ligase complex). Subsequent to DNA damage recognition, both subpathways merge into the same pathway, characterized by damage verification via XPA, DNA unwinding by the helicases XPB and XPD, excision of the damaged DNA fragment by the nucleases excision repair cross‐complementing repair deficiency complementation group 1 (ERCC1)/XPF and XPG, and DNA resynthesis and ligation via Pol δ/ε and ligase I/III. Specifically, it has been reported that UV‐C light activates cellular PARylation 11, and that PARP inhibition renders cells sensitive to UV‐C irradiation 13 and sensitizes mice for the development of UV‐B‐induced skin cancers 19. With regard to the potential underlying molecular mechanisms of these findings, several recent studies indicated that the damage recognition protein DDB2 directly interacts with PARP1, which promotes PARP1 activation, subsequent chromatin relaxation, and recruitment of XPC and the chromatin‐modifier ALC1 12. Furthermore, it has been reported that PARP1 physically interacts with XPA and PARylation may strengthen this interaction 17. XPA is considered the rate‐limiting factor in the NER process. It is part of the core NER incision complex that is essential for the initial phase of the NER and interacts with DNA, as well as with many other NER subunits, including the UV–DDB complex 20. Mechanistically, it is considered that XPA contributes to DNA damage verification and the coordination of assembly of downstream NER complexes 20. Using peptide binding studies, Pleschke *et al*. 22 identified a PAR‐binding motif (PBM) within XPA consisting of a consensus sequence of basic and hydrophobic aa 22. Follow‐up studies using an electrophoretic mobility shift assay (EMSA) and surface plasmon resonance analysis revealed that also full‐length XPA interacts with PAR with high affinity 23. Interestingly, XPA displayed differential binding for PAR depending on the polymer chain length, thus favoring binding to long PAR chains (55‐mer) over short ones (16‐mer) 23. These results suggest a regulatory function of PARylation for XPA activity during NER.

In the present study, we address the functional consequences of the noncovalent XPA–PAR interaction. In particular, we show that the XPA‐PBM is highly conserved within the animal kingdom and that the XPA–PAR interaction is mediated by specific basic amino acids. Furthermore, we demonstrate that, although the DNA binding affinity of XPA is inhibited by noncovalent PAR binding, XPA, in turn, strongly stimulates PARP1 activity in an *in vitro* PARylation assay. In addition, cellular studies revealed that PARylation is necessary for efficient recruitment of XPA to sites of laser‐induced DNA damage. Taken together, our results reveal that XPA, PARP1 and PAR regulate each other's functions in a tightly controlled manner, presumably to fine‐tune the spatio‐temporal assembly or disassembly of macromolecular complexes during NER.

## Results

### PARylation affects cellular NER efficacy

**Figure 1:**
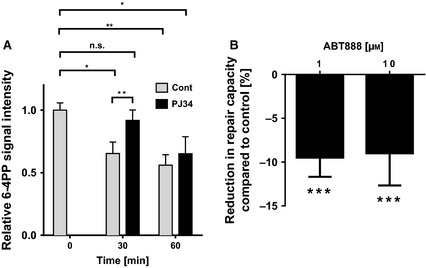
PARP inhibition decreases cellular NER capacity. (A) Removal of 6‐4PP in HeLa cells after UV‐C irradiation (± PARP inhibition, PJ34, 5 μm). Cells were irradiated with 10 J·m^−2^ UV‐C light, DNA was extracted at time points as indicated, and equal amounts (250 ng per slot) were immobilized on a nylon membrane. Afterwards, 6‐4PPs were detected using an anti‐6‐4PP mAb. Data represent the mean ± SEM from five independent experiments, each performed in technical triplicates. Statistical analysis was performed using one‐way analysis of variance testing followed by Dunnett's post‐test. Comparison between control and PJ34‐treated samples at 30‐min repair time was performed using a two‐tailed paired *t*‐test. (B) NER capacity in primary human fibroblasts was measured by host cell reactivation assay as described previously 53. ABT888 was added to the medium 30 min before transfection of cells with an UV‐C‐irradiated reporter plasmid. Repair capacity was evaluated 24 h after transfection by analyzing expression levels of fluorescence reporter proteins using flow cytometry. Data represent the mean ± SEM from four independent experiments. Statistical analysis was performed using the Wilcoxon signed rank test. *P < 0.05, **P < 0.01, ***P < 0.001.

To determine whether PARylation plays a functional role in NER, we used two independent approaches to study cellular NER capacity as a function of cellular PARylation. First, we measured the kinetics of 6‐4PP removal in HeLa cells after UV‐C irradiation using an immunochemical slot blot technique. As shown in Fig. 1A, after a recovery time of 30–60 min, the signal intensities of 6‐4PP immunostaining decreased significantly by approximately 35–45%, indicating the efficient removal of these adducts in HeLa cells. Of note, removal of 6‐4 photoproducts occurred at a significantly slower rate in the presence of the PARP inhibitor PJ34 (Fig. 1A), which is in agreement with previous studies 12. Because it has been revealed that PJ34 exhibits significant PARP‐independent off‐target effects 25, we used the clinically relevant pharmacological PARP inhibitor ABT888 in a second approach to investigate the role of PARylation in NER. Accordingly, we performed a fluorimetric host cell reactivation assay in human primary foreskin fibroblasts based on two‐wavelength enhanced green fluorescent protein (eGFP) and DsRed reporter technology 27. In this assay, a plasmid encoding a DsRed reporter protein was irradiated with UV‐C light. Then, the damaged plasmid was cotransfected with a non‐irradiated plasmid encoding eGFP, which served as a transfection control, into fibroblasts. Cells were analyzed by flow cytometry, and restoration of dsRed expression was used as a direct readout for cellular NER capacity. Figure 1B demonstrates that PARP inhibition led to a mild but significant reduction in the repair capacity by approximately 10%, which is in agreement with previous results comparing wild‐type with XPA‐deficient or PARP1‐depleted fibroblasts 13. In summary, our results confirm a moderate but significant role of PARylation in the repair of UV‐C‐induced DNA damage.

### XPA interacts with PAR noncovalently via a strongly conserved PBM

**Figure 2:**
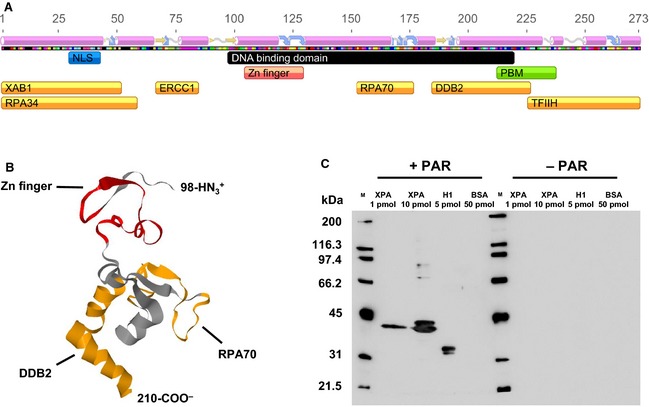
XPA binds PAR in a noncovalent manner. (A) Functional domains of XPA including predicted secondary structure elements (α‐helices in pink; β‐strands in blue; disordered in yellow). The PBM is indicated in green, the Zinc finger domain (Zn finger) in red, the DNA binding domain in black, the nuclear localization signal (NLS) in blue, and protein–protein interaction domains in orange. XAB1 indicates XPA binding protein 1; RPA, replication protein A. (B) NMR model of the minimal DNA‐binding fragment of human XPA (amino acids 98–210) based on PDB file ‘1XPA’ 36. The Zn finger domain is highlighted in red; the RPA70 and DDB2 interaction domains in orange. The PBM is located right next to the C‐terminal end of the structure shown and was not part of the analyzed protein fragment. (C) PAR overlay blot demonstrating noncovalent XPA–PAR interaction. Proteins (in amounts as indicated) were separated by SDS/PAGE, immobilized on a nitrocellulose membrane, and incubated without or with 0.2 μm PAR. After high‐salt washing of membranes, protein‐bound PAR was detected using the anti‐PAR mAb 10H. PAR binding to histone H1 served as a positive control; absence of PAR binding to BSA served as a negative control.

**Figure 3:**
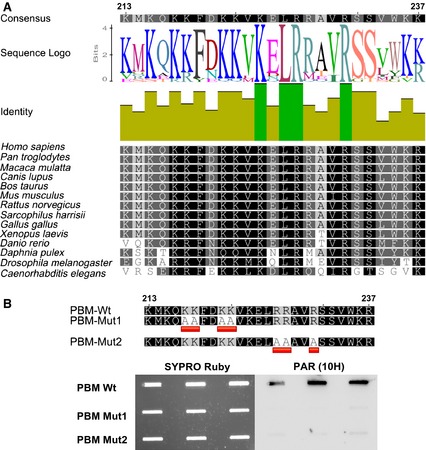
The PBM of XPA is highly conserved and the XPA–PAR interaction is mediated by basic amino acids. (A) Sequence alignment of the PBM of XPA of various animal species as indicated. Amino acids with similar biochemical properties are indicated by the same gray‐shade. (B) Sequence alignment and PAR overlay blot with XPA peptides comprising amino acids of the wild‐type PBM and two mutant forms with exchanges of basic amino acids to alanines as indicated. Peptides were immobilized on a nitrocellulose membrane and incubated with 0.2 μm PAR. After high‐salt washing, PAR binding was detected using the anti‐PAR monoclonal antibody 10H. SYPRO Ruby staining served as a peptide loading control. Both PBM mutants show strongly diminished PAR binding.

Using EMSA and surface plasmon resonance analyses, we previously demonstrated that full‐length XPA displays a high‐affinity binding to long PAR chains (55‐mer), with *K*_D_ values in the low nanomolar range 23. Furthermore, Pleschke *et al*. 22 mapped the PAR‐binding site to a consensus sequence of basic and hydrophobic amino acids (amino acids 213–237). Interestingly, this sequence overlaps with the DNA binding‐, DDB2‐ and TFIIH interaction domains of XPA, suggesting that PAR binding controls XPA–DDB2/TFIIH, as well as XPA–DNA interactions (Fig. 2A,B). In the present study, we confirmed the noncovalent XPA–PAR interaction using an alternative approach based on SDS/PAGE separation of full‐length recombinant proteins and subsequent far‐western analysis, by incubating membranes with *in vitro* synthesized PAR, followed by high‐salt washing to disrupt unspecific binding, and immunochemical detection of noncovalently bound PAR (‘PAR overlay assay’). Figure 2C demonstrates that PAR bound to XPA with an affinity similar to that observed for the prototypical PAR‐binding protein histone H1. The multiple bands of XPA in the lane with 10 pmol XPA loaded are assumed to reflect distinct conformations of the protein 21. Next, using a peptide approach, we confirmed the binding of PAR to the consensus sequence identified by Pleschke *et al*. 22 (Fig. 3). Interestingly, when aligning the PBM sequences of 14 animal species with known or predicted PARylation activity, a high level of homology within the PBM sequence is observed (Fig. 3A). Of note, three basic amino acids and one hydrophobic amino acid are completely conserved across all species analyzed, suggesting a biological relevant function of the PBM. To determine which amino acids are necessary for the XPA–PAR interaction, we exchanged four highly conserved basic amino acids in the N‐terminal part (PBM‐Mut1) or three amino acids in the C‐terminal part (PBM‐Mut2) to alanines. PAR overlay studies revealed that both mutants exhibit strongly diminished PAR‐binding affinities, indicating that basic amino acids are of paramount importance for the XPA–PAR interaction (Fig. 3B). In summary, in the present study, we extended our investigation into the characteristics of noncovalent XPA–PAR interaction, validating the interaction of PAR with full‐length XPA, as well as peptides comprising the XPA–PBM, and demonstrating that the XPA–PAR interaction depends on the presence of several highly conserved basic amino acids.

### Mutual functional regulation of XPA and PARP1

**Figure 4:**
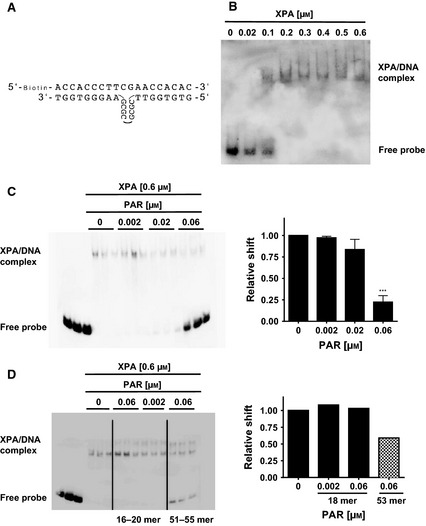
XPA–PAR interaction inhibits binding of XPA to DNA *in vitro*. EMSA to assess the effect of PAR on XPA–DNA interaction. XPA was pre‐incubated with increasing concentrations of PAR as indicated and then an EMSA with a DNA substrate of XPA was performed. (A) A biotinylated oligonucleotide (19‐mer) was annealed with an unlabeled oligonucleotide (25‐mer), which leads to loop formation serving as an XPA binding substrate 52. (B) XPA–DNA EMSA with 200 fmol of the DNA substrate shown in (A) and increasing concentrations of recombinant XPA as indicated. Reaction mixtures were separated by Tris/borate/EDTA‐PAGE, DNA–XPA complexes were immobilized on a nylon membrane, and bound DNA detected via streptavidin‐HRP. (C) Left. The presence of long PAR (51–55‐mer) significantly inhibited the formation of an XPA–DNA complex in a dose‐dependent manner. Right: densitometric evaluation of three independent experiments each performed in technical replicates. Data represent the mean ± SEM. Statistical evaluation was performed using one‐way analysis of variance testing followed by Dunnett's post‐test. ***P < 0.001. (D) Left: short PAR (16–20‐mer) does not affect XPA–DNA interaction. Right: densitometric analysis of two independent experiments each performed in technical triplicates.

PARylation of, or PAR binding to several DNA binding proteins, such as WRN and p53, reduces the DNA binding affinity of these proteins 24. To test whether the noncovalent XPA–PAR interaction affects the DNA binding affinity of XPA, we performed EMSA studies analyzing the binding of recombinant XPA protein to a known DNA binding substrate containing a looped structure on one strand of the duplex oligonucleotide (Fig. 4A,B). Intriguingly, when pre‐incubating XPA with increasing amounts of PAR of a defined chain length of 51–55 ADP‐ribose moieties, a significant reduction in the DNA binding affinity of XPA was observed (Fig. 4C). In line with our previous results showing that XPA favors the binding of longer PAR chains compared to short ones 23, only long PAR (51–55‐mer) significantly inhibited XPA–DNA binding (Fig. 4C), whereas short PAR (16–20‐mer) did not affect the XPA–DNA interaction (Fig. 4D). These results clearly indicate that the noncovalent XPA–PAR interaction directly affects the functionality of XPA.

**Figure 5:**
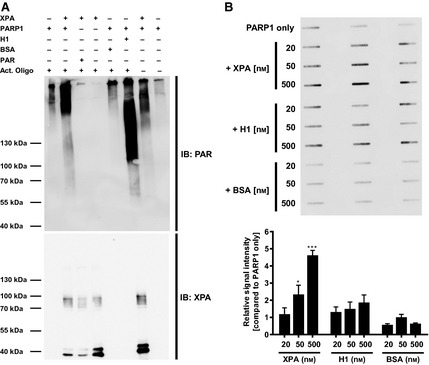
XPA stimulates PARP1 activity *in vitro*. (A) PARylation assay using recombinant PARP1 and XPA in the presence of NAD^+^ and an activator oligonucleotide mimicking DNA strand breaks. Equal amounts of reaction mixtures were separated by SDS/PAGE, immobilized on a nitrocellulose membrane, and stained for PAR and XPA using specific antibodies. Histone H1 served as a positive control; BSA served as a negative control. (B) Semi‐quantitative slot‐blot PARylation assay using equal amounts of recombinant PARP1 and increasing amounts of XPA, H1 and BSA. Top: representative membrane stained for PAR using the mAB 10H (in technical triplicates). Bottom: densitometric evaluation of three independent experiments each performed in technical triplicates. Data represent the mean ± SEM. Statistical analysis was performed using one‐way analysis of variance followed by Dunnett's post‐test. *P < 0.05, ***P < 0.001.

Because it has been suggested that DDB2 stimulates PARP1 activity and it is known that other PARylated proteins such as histone H1 stimulate PARP1 activity, we tested whether XPA also directly influences PARP1 activity. Accordingly, we tested PARP1 activity using two different approaches. First, we performed an *in vitro* PARylation assay using recombinant PARP1 and XPA in the presence of NAD^+^ and an activator oligonucleotide that simulates DNA strand breaks and therefore serves as a strong PARP1 activator (Fig. 5A). In this assay, the reaction is carried out for 15 min in the presence of high concentrations of NAD^+^ and therefore detects the maximum PAR formation activity under equilibrium conditions. As expected, histone H1, but not BSA, which served as a negative control, activated PARP1. Interestingly, XPA strongly stimulated PARP1 activity, suggesting a mutual regulation of PARP1–XPA protein function in the context of active PARylation activity. By contrast to the noncovalent XPA–PAR interaction, covalent protein modification is expected to withstand SDS/PAGE sample preparation conditions. Because no clear PAR signals were detected in the molecular mass range of monomeric (approximately 40 kDa) or dimeric XPA (approximately 80 kDa), we conclude that covalent PARylation of XPA plays no or only a minor role in the regulation of XPA by PAR. Although, we cannot fully exclude any potential residual DNA contamination of our recombinant XPA preparation, it is interesting to note that XPA stimulated PARP1 activity even in the absence of activator DNA, suggesting direct activation of PARP1 based on protein–protein interaction. Next, we used a slot‐blot PARylation assay to analyze the stimulatory activity of XPA. In this assay, the PARylation reaction was carried out only for 30 s in the presence of excess amounts of activator DNA and therefore determines the PARP1 activity in the dynamic phase of the reaction. As a result of its higher throughput, the results from this assay can be assessed in a semi‐quantitative manner. As shown in Fig. 5B, these experiments confirmed that XPA stimulates PARP1 activity in a dose‐dependent manner. Remarkably, under the conditions used in this assay, stimulation was even higher than that achieved by histone H1. In summary, our results from EMSA XPA–DNA binding studies and from *in vitro* PARylation assays reveal a reciprocal regulation of PARP1 and XPA protein function, suggesting that the physical and functional regulation of the two factors contributes to efficient DNA repair in the cell.

### PARP inhibition alters the recruitment kinetics of XPA to sites of laser‐induced DNA damage

**Figure 6:**
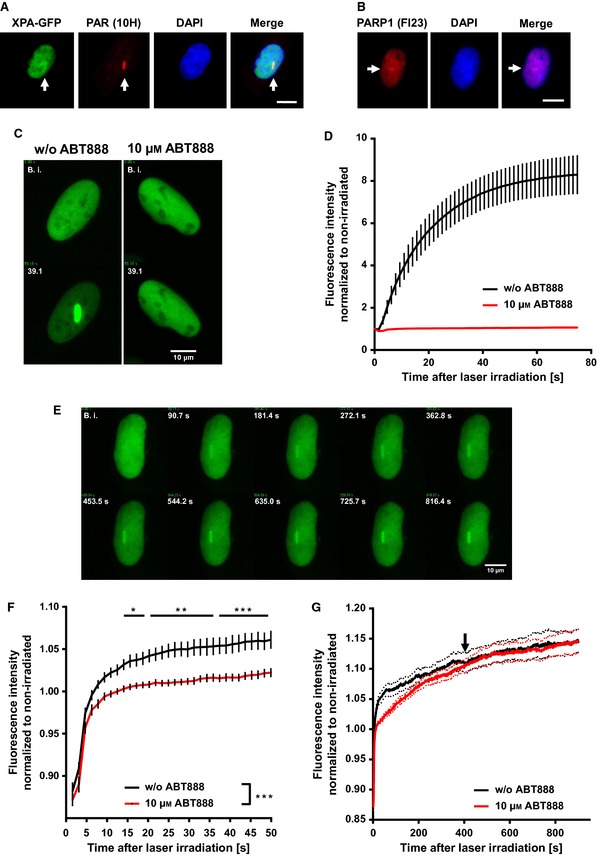
PARP inhibition impairs recruitment of XPA‐eGFP to sites of laser‐induced DNA damage. Nuclei of U2OS cells were irradiated with a pulsed femtosecond laser as described in the Materials and methods. (A) Immunofluorescence microscopic analysis of XPA‐eGFP‐transfected cells demonstrating the local formation of PAR at approximately 1 min post irradiation. Cells were fixed with PFA and stained for PAR using the mAB 10H. (B) Immunofluorescence microscopic analysis demonstrating the recruitment of PARP1 to sites of laser‐induced DNA damage at approximately 4 min post irradiation. Cells were fixed with PFA and stained for PARP1 using the mAB FI23. (A, B) Images are brightness and contrast‐adapted for better visibility. (C, D) Accumulation of XRCC1‐eGFP at sites of laser irradiation and its inhibition by PARP inhibitor treatment (ABT888). (C) Snapshots of cell nuclei before irradiation and 39 s post irradiation in the absence or presence of 10 μm ABT888. (D) Recruitment kinetics of XRCC1‐eGFP to sites of laser‐induced DNA damage, acquired for 75 s in the absence or presence of 10 μm ABT888. ABT888 completely blocked the recruitment of XRCC1 to sites of laser‐induced damage. Data represent the mean ± SEM from six cells per condition. These results are in agreement with previous data 31. (E–G) Accumulation of XPA‐eGFP at sites of laser irradiation in the absence and presence of ABT888. (E) Time series of XPA‐eGFP accumulation at sites of irradiation at different time points as indicated (without PARP inhibition). (F) Short‐term recruitment kinetics of XPA‐eGFP were acquired in the absence or presence of 10 μm ABT888. (G) Long‐term recruitment studies of XPA‐eGFP. Data represent the mean ± SEM of ≥ 10 cells analyzed. Statistical evaluation was performed using two‐way analysis of variance followed by Sidak's post‐test. *P < 0.05, **P < 0.001, ***P < 0.001. Scale bar = 10 μm.

To determine whether PARylation affects the spatio‐temporal localization of XPA at sites of DNA damage, we transfected U2OS cells with a GFP‐XPA expression construct and analyzed the recruitment kinetics of XPA via laser scanning microscopy (Fig. 6). In this experimental set‐up, we used a pulsed femtosecond laser at a wavelength of 775 nm to induce DNA damage. As described previously with a similar system 29, this procedure results in multiphoton absorption in the confocal volume, thereby mimicking light of shorter wavelength analogous to the wavelength of 258 nm that efficiently induces DNA damage, including 6‐4PP and CPDs 29. Moreover, PAR and PARP1 are present at sites of laser irradiation as evaluated by immunofluorescence microscopy using PAR and PARP1 specific antibody staining after laser irradiation and paraformaldehyde (PFA) fixation of cells (Fig. 6A,B). To test the influence of PARylation on XPA recruitment kinetics, we incubated U2OS cells with the PARP inhibitor ABT888 (10 μm). PARP inhibition was highly effective in preventing the accumulation of XRCC1 at damaged sites, as reported previously 31, thus validating the functionality of the system and the reagents used (Fig. 6C,D). As is evident from Fig. 6E, laser irradiation of U2OS cells triggered the recruitment of XPA to sites of irradiation. Quantitation of these results revealed a biphasic recruitment pattern of XPA to sites of laser‐induced DNA damage. An initial very fast recruitment of XPA to irradiated sites occurred within < 10 s. Subsequently, a slower steady increase of the GFP signal was observed until the end of measurements after 15 min (Fig. 6F,G). Interestingly, PARP inhibition also significantly decreased the XPA‐GFP signal intensity at damaged sites, particularly in the early phase of XPA recruitment. After approximately 6.8 min post irradiation, the signal intensities of XPA‐GFP with or without ABT888 treatment leveled up (Fig. 6G, black arrow), indicating that PARylation facilitates the recruitment of XPA to sites of DNA damage; however, it does not influence the abundance of XPA at the lesions in later phases of NER. These findings provide direct evidence for an active role of PARylation in the spatio‐temporal control of cellular XPA localization during the DNA damage response in the cell.

## Discussion

**Figure 7:**
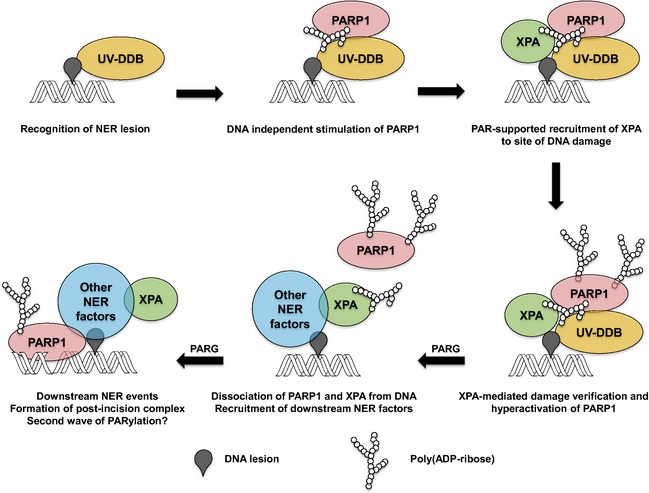
Simplified model summarizing the potential sequence of events during the formation of the NER pre‐incision complex. The model is based on findings from the present study and previous studies 11. It is important to note that many of these processes are highly dynamic (e.g. PARG, which exhibits PAR endo‐ as well as exonuclease activity, becomes activated immediately after PARylation, is induced, and triggers PAR degradation and release of ‘free’ PAR). Moreover, other factors (e.g. XPC) are involved in the formation of the pre‐incision complex, which have been omitted in the model for clarity. A second wave of PARylation may be associated with the incision of DNA by NER endonucleases, which can lead to DNA‐dependent activation of PARP1. For details, see Discussion.

PARylation is a drastic post‐translational modification that leads to the spatio‐temporal control of protein function and subcellular localization 3. PARylation comes in two flavors. On the one hand, proteins can be covalently modified by PAR at specific amino acids, whereas, on the other hand, PAR can bind to target proteins noncovalently via at least five different PAR‐binding modules 7. Of the latter, the PBM, which is a loosely conserved sequence of approximately 20 amino acids containing a cluster rich in basic and hydrophobic amino acids, is the most widespread one, because it is present in hundreds of cellular proteins 22. Approximately 50 of them have been validated experimentally to bind PAR in a noncovalent manner 7. Previously, Pleschke *et al*. 22 identified a PBM in the key NER factor XPA and, in a follow‐up study, we characterized biochemical properties of the noncovalent interaction of PAR with full‐length recombinant XPA 23. In the present study, we addressed the functional consequences of this interaction (Fig. 7). First, we verified previous results indicating that PARylation plays a significant role during UV‐C‐induced DNA damage response (Fig. 1) 12. Second, we validated the noncovalent interaction of PAR with full‐length XPA, as well as with a peptide comprising the PBM amino acid sequence, using far‐western‐based PAR overlay assays and showed that several highly conserved basic amino acids are important in mediating this interaction (Figs. 2 and 3). Third, we demonstrated that, on the one hand, noncovalent XPA–PAR binding impaired the binding ability of XPA to a DNA substrate (Fig. 4), whereas, on the other hand, XPA itself stimulates PARP1 activity in a reciprocal manner (Fig. 5). Finally, laser‐induced recruitment studies indicate that PARylation actively controls the spatio‐temporal localization of XPA in the cell (Fig. 6).

The localization of the PBM within the XPA sequence suggests a functional role of PAR binding in coordinating the interaction of XPA with other macromolecules because it is localized right at the interface of the minimal DNA binding and DDB2 binding domains to its N‐terminal side 20, as well as the TFIIH binding domain to its C‐terminal side 37. Therefore, it is possible that the XPA–PAR interaction induces conformational changes in the protein structure or that PAR serves as a repulsive or adhesive factor for protein–protein interactions. Whether PAR supports or disrupts protein–protein interactions most likely depends on the specific complex that is targeted. In particular, the proximity of the DDB2 interaction domain is of high interest because XPA and DDB2 share some obvious similarities with respect to their regulation by PARylation. Thus, both XPA and DDB2 directly interact with PARP1 in cells 12, as well as with PAR 12. Moreover, both proteins directly stimulate PARP1 activity 12. The finding that DDB1 is a PARylation target as well 39 further supports a functional role of PARylation in the regulation of the UV–DDB complex. A conceivable sequence of events occuring during the formation of the NER pre‐incision complex, which could reconcile the above findings, can be proposed (Fig. 7):

- The UV‐DDB complex binds to DNA photolesions, which attracts and activates PARP1.
- Production of PAR then leads to an initial opening of the chromatin structure and recruitment of downstream factors, such as ALC1 and XPC 15. Interestingly, XPC can be also covalently PARylated upon induction of genotoxic stress 9 and possesses a putative PBM 34. In this context, it has been recently demonstrated that PARylation actively supports the recruitment of XPC to sites of UV damage 12. These results resemble our findings showing that inhibition of PARylation impairs the recruitment of XPA to sites of laser‐induced damage, which provides direct evidence that PAR also supports the recruitment of XPA to the site of DNA damage. From our XPA‐GFP recruitment studies, it becomes evident that PARylation is of particular importance in the early phase of XPA recruitment; however, it is not essential in the long term. This is consistent with our DNA repair studies, which demonstrate that PARP inhibition impairs NER activity but is unable to completely block the repair of UV‐C‐induced damage. Whether or not such a positive effect of PARylation on the recruitment of XPC and XPA is caused by a direct attraction of the proteins to PAR or by rendering the site of the damage more accessible by an opening to the chromatin structure is as yet unknown, although it is likely that both factors contribute the observed effects.
- Subsequently the direct XPA–PARP1 interaction 17 can further stimulate PARP1 activity, as suggested by our *in vitro* observations, which may trigger additional PARylation and further orchestrate changes in chromatin structure and the composition of the NER complex. In this regard, an interesting question would be what actually triggers the activation of PARP1 at the sites of DNA damage because all these steps noted above occur before the induction of strand breaks by the endonucleases XPG and ERCC1/XPF and the formation of the post‐incision complex. Therefore, it is likely that direct protein–protein interactions of DDB2 and XPA are sufficient to stimulate PARP1 activity, even without binding of PARP1 to DNA strand breaks. Our results showing that XPA stimulates PARP1 activity even in the absence of activator DNA, as well as previous reports demonstrating that PARP1 can be activated by direct protein–protein interactions or post‐translational modifications 41, support such a scenario.
- Next, based on the finding that PAR inhibits the XPA–DNA interaction *in vitro*, it is tempting to speculate that, in the course of the NER process, the XPA–PAR interaction causes the disassembly of XPA from its DNA substrates.
- Apart from its role in the pre‐incision complex, PARylation is likely to play a role in the orchestration of the post‐incision complex because a second wave of PARylation has been observed at later stages during UV‐induced DNA damage response 11. The significance of this finding has yet to be addressed in future studies.

Another layer of complexity is added by the fact that XPA and PARP1 are reciprocally regulated by the deacetylase SIRT1. In general, the functions of PARP1 and SIRT1 are strongly linked: first, by competing for the same substrate (i.e. NAD^+^) and by being inhibited by the same by‐product of catalysis (i.e. nicotinamide) and, second, by physically interacting and modifying each other, thereby reciprocally regulating their activities 42. In addition, deactylation of XPA by SIRT1 is required for optimal NER functionality and enhances the interaction of XPA with replication protein A 44. The molecular details and dynamics of this XPA–PARP1–SIRT1 network are far from understood and further studies are needed to determine how these factors interact and react specifically during cellular NER.

Interestingly, two polymorphisms (i.e. Arg228Gln and Val234Leu), which are located within the XPA–PBM sequence, are associated with an improved repair of benzo(a)pyrene‐induced DNA lesions 45. In addition, another study reported that PARP1 deficiency renders cells more sensitive to benzo(a)pyrene treatment 46. Therefore, it will be interesting to determine whether the XPA–PAR interaction is altered by the introduction of the two polymorphisms noted above. Interestingly, both polymorphisms had minimal or no effect on the repair of UV‐induced DNA damage 47, suggesting that PARylation controls XPA and NER in a lesion‐specific manner, and therefore may represent an important mechanism to fine‐tune the repair of different NER substrates. Moreover, PARP1 has a well‐documented role in other DNA repair mechanisms, such as base excision and DNA single‐strand break repair, as well as DNA double‐strand break repair. This raises the hypothesis that PARylation orchestrates the interplay, dynamics, and ranking of priority of the different DNA repair pathways to repair the different types of heterogeneous DNA damage lesions that are localized in close proximity to each other in the genome. During these processes, a tight spatio‐temporal regulation of the production and degradation of PAR would be of central importance. Therefore, in addition to studying activation of PARPs and the occurrence of PAR at the site of the damage, it will be necessary to address the role of the PAR degrading enzyme PARG in more detail. Interestingly, a similar reciprocal regulation has been shown for PARP1 and the DNA glycosylases Ogg1 and Neil1. Both proteins physically interact with PARP1, which leads to the stimulation of PARylation activity, whereas activated PARP1 inhibits the incision activities of Ogg1 and Neil1 48.

In conclusion, the present study reveals that PARylation supports cellular NER efficacy and suggests that this is at least in part mediated by a reciprocal regulation of XPA and PARP1 functions. Therefore, our results, when considered together with previous studies reporting a role for PARylation in the control of other NER factors, such as the UV–DDB complex and XPC, support a scenario in which PARylation acts as an important fine‐tuning mechanism in the spatio‐temporal control of protein function and localization during cellular NER. It will be worthwhile to investigate exactly how PARylation influences the interplay of the various NER factors at specific sites of DNA damage and to determine whether PARylation is a regulatory factor for coordinating the repair of multiple DNA lesions in close proximity by different DNA repair mechanisms.

## Materials and methods

### Cell culture

HeLaS3 cells were cultured in DMEM (41966; Gibco, Gaithersburg, MD, USA) supplemented with 10% fetal bovine serum (Biochrom Ltd., Cambridge, UK), 100 U·mL^−1^ penicillin and 100 μg·mL^−1^ streptomycin (Gibco) and 2 mm l‐Gln (Gibco) in 5% CO_2_ at 37 °C. U2OS cells were cultured in McCoy's 5A (modified) medium (26600; Gibco) supplemented with fetal bovine serum and penicillin and streptomycin as above. High Five and *SF*9 insect cells were grown at 27 °C in TNM‐FH medium (Bio&SELL, Feucht, Germany) supplemented with penicillin and streptomycin, as indicated above.

### Recombinant proteins

Tag‐free PARP1 and His‐tagged XPA were overexpressed in *SF*9 and High Five cells, respectively, using the baculovirus system. PARP1 overexpression and purification was carried out as described previously 23. For XPA purification, High Five cells, infected with an XPA expression virus at a multiplicity of infection of 4 for 72 h, were lysed for 20 min in XPA lysis buffer (50 mm potassium phosphate buffer, pH 8.0; 100 mm KCl; 0.5% NP‐40; 1 mm EDTA; 10% glycerol; 0.5 mm phenylmethanesulfonyl fluoride) followed by sonication for 10 min at 4 °C. The cell lysate was centrifuged for 1 h at 47 000 ***g*** and then the supernatant was applied on a Ni‐NTA Superflow column (Novagen, Madison, WI, USA) that had been equilibrated in XPA purification buffer (100 mm KCl, 0.01% NP‐40, 1 mm EDTA, 10% glycerol, 0.5 mm phenylmethanesulfonyl fluoride). The column was washed with 1 mm imidazole and recombinant XPA eluted with purification buffer supplemented with 100 mm imidazole. The protein was dialyzed and stored in 25 mm Hepes‐KOH (pH 7.8), 100 mm KCl, 10% glycerol and 1 mm EDTA at −80 °C.

### *In vitro* synthesis and HPLC fractionation of PAR

PAR synthesis and HPLC fractionation was performed as reported by Kiehlbauch *et al*. 51 with modifications as described previously 23. Briefly, 75 nm of recombinant PARP1 was incubated in a mixture containing 100 mm Tris‐HCl (pH 7.8), 10 mm MgCl_2_, 1 mm dithiothreitol, 60 μg·mL^−1^ histone H1, 180 μg·mL^−1^ histone H2a, 50 μg·mL^−1^ double‐stranded activator oligonucleotide (5′‐GGAATTCC‐3′) and 1 mm β‐NAD^+^ for 20 min at 37 °C. The reaction was stopped by adding ice‐cold trichloroacetic acid (TCA) to a final concentration of 10% (w/v). After precipitation and centrifugation at 9000 ***g*** for 10 min at 4 °C, the pellet was washed twice with ice‐cold ethanol. PAR was detached from proteins by incubation in 0.5 m KOH and 50 mm EDTA for 10 min at 37 °C. After adjustment to pH 7.5, DNA and proteins were digested by 200 μg·mL^−1^ DNase I and 100 μg·mL^−1^ proteinase K. PAR was finally purified by phenol–chloroform extraction and ethanol precipitation. Size‐fractionation of PAR was performed with an Agilent 1100 HPLC system (Agilent Technologies Inc., Santa Clara, CA, USA) using a semi‐preparative DNA Pac PA100 column (Dionex Corp., Sunnyvale, CA, USA). PAR fractions were eluted using a multistep NaCl gradient in 25 mm Tris‐HCl (pH 9.0), ethanol‐precipitated, dissolved in water, with the concentration determined by measuring *A*_258_, and characterized on a silver‐stained sequencing gel (GELCODE Color silver stain; Pierce Biotechnology, Rockford, IL, USA).

### PAR overlay assays

To analyze XPA–PAR interactions, PAR overlay assays were performed as described previously 24 with some modifications. Briefly, for overlay assays using recombinant proteins, i.e., histone H1, BSA and XPA, in amounts as indicated, were subjected to 12% SDS/PAGE and transferred by wet blotting onto a nitrocellulose membrane (GE Healthcare, Little Chalfont, UK). Then, the membrane was incubated overnight at 4 °C either in TBST (150 mM NaCl, 10 mM Tris pH 8, 0.05% Tween 20) or in TBST containing 0.2 μm PAR. The next day, the membrane was blocked in TBST supplemented with 5% (w/v) skim milk, incubated in anti‐PAR serum (10H, a kind gift from M. Miwa, Nagahama Institute of Bioscience and Technology, Japan, and T. Sugimura, National Cancer Center Research Institute, Tokyo, Japan,) or anti‐XPA serum (FL‐273; Santa Cruz Biotechnology, Santa Cruz, CA, USA) solutions, repeatedly washed in TBST, and incubated for 1 h in goat‐anti‐mouse‐horseradish peroxidase (HRP) or goat‐anti‐rabbit‐HRP (dilution 1 : 2000; Dako, Glostrup, Denmark) solutions, respectively. Subsequently, blots were washed and chemiluminescence detected with ECL Advance (GE Healthcare) using a LAS‐4000 chemiluminescence detector (GE Healthcare). For peptide studies, peptides harboring the XPA PAR‐binding‐site (PBM‐wild‐type, amino acids 213–237) and its mutant forms [PBM‐Mut1 (K217A, K218A, K221A, K222A) and PBM‐Mut2 (R227A, R228A, R231A)] were custom‐synthesized (GenScript Corporation, Piscataway, NJ, USA). Peptides were dissolved in 10% acetic acid, 15 pmol were immobilized on a nitrocellulose membrane via slot blotting, and membranes were air dried for 1 h. Testing for PAR‐binding ability was performed as described for proteins. To ensure that equal amounts of peptides were loaded, SYPRO Ruby protein staining (Molecular Probes, Carlsbad, CA, USA) was performed in accordance with the manufacturer's instructions.

### PARP activity measurements

PARP1 activity was determined using a slot blot assay as described previously with some modifications 50. Briefly, 5 nm PARP1 was pre‐incubated with or without XPA, H1 or BSA (20, 50 or 500 nm) in reaction buffer [100 mm Tris‐HCl, pH 7.8, 10 mm MgCl_2_, 1 mm dithiothreitol, 25 μg·mL^−1^ double‐stranded activator oligonucleotide (5′‐GGAATTCC‐3′)] for 15 min on ice. The reaction was started by adding 200 μm NAD^+^ and carried out for 30 s at 30 °C. The reaction was stopped by adding TCA to a final concentration of 10% (w/v) and then samples were vacuum‐aspirated onto a nylon membrane (GE Healthcare). PAR was immobilized by incubating the membrane for 1.5 h at 95 °C. The membrane was blocked for 1 h in TBST supplemented with 5% (w/v) skim milk, incubated for 1 h in anti‐PAR serum solution (10H), repeatedly washed, incubated for 1 h in secondary antibody goat‐anti‐mouse‐HRP solution (dilution 1 : 2000), washed again and chemiluminescence was analyzed. Densitometric quantification of the PAR signal was performed using imagej (NIH, Bethesda, MD, USA). Analysis of PARP1 activity via western blotting was performed based on the protocol used for activity measurements via slot‐blotting with some modifications: reactions were carried out in the presence of 100 nm PARP1 and 200 nm XPA, 150 nm H1 or 100 nm BSA after the addition of 1 mm NAD^+^ for 15 min. Reactions were stopped by adding TCA to a final concentration of 10% (w/v). Samples were subjected to 8% SDS/PAGE and proteins were transferred by wet blotting onto a nitrocellulose membrane and PAR and XPA (FL‐273; Santa Cruz Biotechnology) were detected by immunochemical staining as described above.

### EMSA

To analyze XPA–DNA interactions, a loop‐containing oligonucleotide was used as a XPA binding substrate as described previously 52 with modifications. Briefly, recombinant XPA was pre‐incubated for 30 min with PAR (of defined chain length and concentration as indicated) in EMSA binding buffer (25 mm Hepes‐KOH, pH 8.3; 30 mm KCl; 0.4 mm MgCl_2_; 1 mm EDTA; 10% glycerol; 45 μg·mL^−1^ BSA; 0.9 mm dithiothreitol) before adding 20 nm of the biotinylated duplex oligonucleotide. Samples were incubated for 30 min and then subjected to Tris/borate/EDTA‐PAGE for separation of free and XPA‐bound DNA. The gel was semidry blotted onto a nylon membrane, DNA was immobilized by incubating the membrane for 1.5 h at 95 °C. Unspecific binding sites were blocked by incubating the membrane in TBST supplemented with 5% (w/v) skim milk. Then, the membrane was washed three times in TBST, incubated in streptavidin‐HRP (dilution 1 : 2500; GE Healthcare), washed again and chemiluminescence was analyzed. Quantitative analysis of the band shift was performed using imagej. Relative band shift was calculated by dividing the XPA‐bound‐DNA signal intensity by the overall signal intensity of the whole lane followed by normalization to control samples with XPA/DNA only.

### Host cell reactivation assay

The host cell reactivation assay was performed as described previously 53. The functional restoration of an UV‐C‐irradiated plasmid was measured to quantify NER capacity. In brief, cultures of subconfluent human foreskin fibroblasts (cultured in DMEM supplemented with 10% fetal bovine serum and 50 μg·mL^−1^ gentamycin) were pre‐incubated without or with ABT888 in concentrations as indicated for 30 min. Then, cells were harvested by trypsination and cell suspensions were divided into two aliquots. The first aliquot was transfected with a plasmid mix containing 3 μg of pEGFP plasmid and 15 μg of pDsRed (preparation ‘1’). The second aliquot was transfected with both plasmids in amounts as indicated above, although the pDsRed plasmid had been irradiated with 5000 J·m^−2^ UV‐C (1800 Stratalinker UV Crosslinker; Stratagene, La Jolla, CA, USA) beforehand (preparation ‘2’). Transfections were carried out with 4‐mm gap cuvettes at 0.32 kV and 500 μF with a GenePulser II (Bio‐Rad, Hercules, CA, USA). After electroporation, fibroblasts were immediately seeded in six‐well plates with or without ABT888. Twenty‐four hours after tranfection, cells were harvested and fluorescence signals analyzed by flow cytometry (FACSCalibur; Becton‐Dickinson Biosciences, Franklin Lakes, NJ, USA). Repair capacity was calculated from the relative amounts of red fluorescent cells compared to green fluorescent cells between preparation ‘2’ and preparation ‘1’ for each treatment. The results were then normalized to the untreated control.

### 6‐4PP repair kinetics

HeLa S3 cells were irradiated using a UV germicidal lamp with a peak wavelength of 254 nm (10 J m^−2^). Cells were incubated for time periods as indicated at 37 °C in DMEM in the presence or absence of PJ34, before harvesting of cells by trypsination. Genomic DNA was extracted using the High Pure PCR Template Preparation Kit (Roche, Basel, Switzerland) in accordance with the manufacturer's instructions. For slot‐blotting, 750 ng of DNA dissolved in 150 μL of TE buffer was incubated for 10 min at 95 °C on a thermomixer at 550 r.p.m. Subsequently, tubes were placed on ice for 15 min and 150 μL of ice‐cold 2 m sodium acetate was added and tubes inverted six times. One‐third of each reaction was pipetted into a slot of the slot blot manifold and samples were vacuum‐aspirated onto a nylon membrane. The membrane was incubated for 1 h at 95 °C and subsequently unspecific binding sites were blocked by incubating the membrane in TBST supplemented with 5% (w/v) skim milk for 1 h at room temperature. Membranes were incubated in an anti‐6‐4PP antibody solution (dilution 1 : 4000; Cosmo Bio, CAC‐NM‐DND‐002, Tokyo, Japan) for 1 h at room temperature, washed three times with TBST for 5 min at room temperature and incubated with goat‐anti‐mouse‐HRP (dilution 1 : 2000) for chemiluminescence detection.

### Recruitment studies

U2OS cells were seeded on μ‐dishes (ibidi, Martinsried, Germany) 1 day before transfection of the XPA‐GFP expression plasmid (pEGFP‐N1::His‐XPA) using Effectene Transfection Reagent (Qiagen, Valancia, CA, USA) in accordance with the manufacturer's instructions. On the day of irradiation, the medium was replaced with phenol‐red free complete medium (DMEM, 31053l; Gibco) and incubated for 1 h at 37 °C and 5% CO_2_. For PARP inhibition, cells were incubated 30 min prior laser irradiation with 10 μm ABT888 (Selleck Chemicals, Houston, TX, USA). Nonlinear excitation was carried out with ultrashort pulses at a wavelength of 775 nm, which was generated via frequency doubling the output of a mode‐locked erbium‐doped fiber laser in a periodically poled MgO:LiNbO_3_ nonlinear‐optical crystal. The laser system was coupled to a confocal microscope (LSM 700; Carl Zeiss, Oberkochen, Germany) and irradiation took place with a pulsed duration time of 300 fs at a repetition rate of 40 MHz. The peak power density in the focus was 72 GW·cm^−2^ (average power: 10 mW). This procedure resulted in multiphoton absorption in the confocal volume, thereby mimicking light of shorter wavelength. In particular, three photons with an energy corresponding to infrared light at a wavelength of 775 nm were absorbed at the same time, effectively summing up to a photon energy analogous to a wavelength of 258 nm, which is in the UV‐C spectral range. Within the nuclei of GFP‐positive cells a 6 μm long line was irradiated for a total time of 3.73 s using an external scanner (Rapp OptoElectronics GmbH, Hamburg, Gemany). As described previously, irradiation at 775 nm results in different types of DNA damage, including DNA photoproducts, such as 6‐4PP and CPD 29. A Zeiss EC‐Plan‐Neofluar 40×/1.3 oil immersion objective was used for near IR irradiation and laser scanning microscopy. Excitation of eGFP and subsequent scanning was carried out using a 488 nm diode laser at open pinhole settings. Automated recording of recruitment kinetics was facilitated using a custom macro for zen software supplied by the Life Imaging Center (LIC, University of Freiburg, German). To confirm the performance of the ultrafast laser system and the efficacy of the ABT888 treatment, XRCC1‐GFP accumulation was analyzed as a control for each XPA‐GFP recruitment experiment. Data analysis was performed using imagej (http://www.bioimaging-center.uni-konstanz.de/image-analysis/imagej-user-macros/). For each time point before and after laser irradiation, background corrected signal intensity was divided by the total fluorescence intensity of the GFP signal in the nucleus deducting the background. Thereafter, data were normalized to the time point before irradiation and the relative changes in fluorescence intensity were calculated.

### Immunochemical staining for PAR and PARP1 fluorescence microscopy

After laser irradiation, cells were fixed in 4% PFA/NaCl/P_i_ (Merck, Darmstadt, Germany) for 20 min at room temperature. μ‐dishes were washed with NaCl/P_i_, and incubated with 50 mm NH_4_Cl/NaCl/P_i_ for 10 min (PAR) or 100 mm glycine for 1 min (PARP1) at room temperature. Cells were washed twice in NaCl/P_i_, permeabilized in 0.5% Triton X‐100/NaCl/P_i_, again washed twice in NaCl/P_i_, and then incubated in blocking solution (1% BSA in NaCl/P_i_) for 30 min (PAR) or 1 h (PARP1) at room temperature. Thereafter, cells were incubated with primary antibodies specific for PAR (mAb 10H) or PARP1 (mAb FI23) for 1 h at 37 °C, subsequently washed three times in NaCl/P_i_, incubated with secondary antibody gαm Alexa546 (Molecular Probes) at 37 °C for 1 h, and again washed three times in NaCl/P_i_. Finally, DNA was counterstained with Hoechst33342 (Invitrogen, Carlsbad, CA, USA) for 5 min at room temperature and samples analyzed using an epifluorescence microscope (Carl Zeiss).

### Sequence alignments and homology searches

Peptide sequence alignments were performed with geneious software (Biomatters Ltd, Auckland, New Zealand) using the Geneious Alignment option with the cost matrix Blosum62. *In silico* searches for PBMs were performed using the PattInProt motif search tool (http://npsa-pbil.ibcp.fr/cgi-bin/npsa_automat.pl?page=npsa_pattinprot.html) with the algorithm [HKR]‐X‐[AVILFWP]‐[AVILFWP]‐[HKR]‐[HKR]‐[AVILFWP]‐[AVILFWP] allowing two mismatches, modified from Pleschke *et al*. 22.

### Statistical analysis

Statistical analyses were performed with graphpad prism software using appropriate statistical tests as indicated. *P* < 0.05 was considered statistically significant.

## Author contributions

JMFF, OP, DG, EFM, AM designed experiments. JMFF, OP, DG, SV, AF performed experiments. JMFF, OP, DG, AM analyzed data. SB, AL, JB, MS, EFM provided essential scientific input and technical expertise. JMFF, AM, AB wrote the paper. OP, DG, SV, AF, SB, AL, JB, MS, EFM edited the manuscript. AM and AB designed the study.

## References

[1] Mangerich A & Bürkle A (2012) Pleiotropic cellular functions of PARP1 in longevity and aging: genome maintenance meets inflammation. Oxid Med Cell Longev 2012, 321653 23050038 10.1155/2012/321653 PMC3459245

[2] Hottiger MO, Hassa PO, Luscher B, Schuler H & Koch‐Nolte F (2010) Toward a unified nomenclature for mammalian ADP‐ribosyltransferases. Trends Biochem Sci 35, 208–219 20106667 10.1016/j.tibs.2009.12.003

[3] Robert I, Karicheva O, Reina San Martin B, Schreiber V & Dantzer F (2013) Functional aspects of PARylation in induced and programmed DNA repair processes: preserving genome integrity and modulating physiological events. Mol Aspects Med 34, 1138–1152 23454615 10.1016/j.mam.2013.02.001

[4] Martello R, Mangerich A, Sass S, Dedon PC & Bürkle A (2013) Quantification of cellular poly(ADP‐ribosyl)ation by stable isotope dilution mass spectrometry reveals tissue‐ and drug‐dependent stress response dynamics. ACS Chem Biol 8, 1567–1575 23631432 10.1021/cb400170b PMC3795969

[5] Barkauskaite E, Brassington A, Tan ES, Warwicker J, Dunstan MS, Banos B, Lafite P, Ahel M, Mitchison TJ, Ahel I*et al* (2013) Visualization of poly(ADP‐ribose) bound to PARG reveals inherent balance between exo‐ and endo‐glycohydrolase activities. Nat Commun 4, 2164 23917065 10.1038/ncomms3164 PMC3741636

[6] Tucker JA, Bennett N, Brassington C, Durant ST, Hassall G, Holdgate G, McAlister M, Nissink JW, Truman C & Watson M (2012) Structures of the human poly (ADP‐ribose) glycohydrolase catalytic domain confirm catalytic mechanism and explain inhibition by ADP‐HPD derivatives. PLoS One 7, e50889 23251397 10.1371/journal.pone.0050889 PMC3519477

[7] Krietsch J, Rouleau M, Pic E, Ethier C, Dawson TM, Dawson VL, Masson JY, Poirier GG & Gagne JP (2013) Reprogramming cellular events by poly(ADP‐ribose)‐binding proteins. Mol Aspects Med 34, 1066–1087 23268355 10.1016/j.mam.2012.12.005 PMC3812366

[8] Min W, Bruhn C, Grigaravicius P, Zhou ZW, Li F, Kruger A, Siddeek B, Greulich KO, Popp O, Meisezahl C*et al* (2013) Poly(ADP‐ribose) binding to Chk1 at stalled replication forks is required for S‐phase checkpoint activation. Nat Commun 4, 2993 24356582 10.1038/ncomms3993

[9] Jungmichel S, Rosenthal F, Altmeyer M, Lukas J, Hottiger MO & Nielsen ML (2013) Proteome‐wide identification of poly(ADP‐ribosyl)ation targets in different genotoxic stress responses. Mol Cell 52, 272–285 24055347 10.1016/j.molcel.2013.08.026

[10] Vyas S, Chesarone‐Cataldo M, Todorova T, Huang YH & Chang P (2013) A systematic analysis of the PARP protein family identifies new functions critical for cell physiology. Nat Commun 4, 2240 23917125 10.1038/ncomms3240 PMC3756671

[11] Vodenicharov MD, Ghodgaonkar MM, Halappanavar SS, Shah RG & Shah GM (2005) Mechanism of early biphasic activation of poly(ADP‐ribose) polymerase‐1 in response to ultraviolet B radiation. J Cell Sci 118, 589–599 15657079 10.1242/jcs.01636

[12] Robu M, Shah RG, Petitclerc N, Brind'Amour J, Kandan‐Kulangara F & Shah GM (2013) Role of poly(ADP‐ribose) polymerase‐1 in the removal of UV‐induced DNA lesions by nucleotide excision repair. Proc Natl Acad Sci USA 110, 1658–1663 23319653 10.1073/pnas.1209507110 PMC3562836

[13] Ghodgaonkar MM, Zacal N, Kassam S, Rainbow AJ & Shah GM (2008) Depletion of poly(ADP‐ribose) polymerase‐1 reduces host cell reactivation of a UV‐damaged adenovirus‐encoded reporter gene in human dermal fibroblasts. DNA Repair (Amst) 7, 617–632 18289944 10.1016/j.dnarep.2008.01.001

[14] Pines A, Mullenders LH, van Attikum H & Luijsterburg MS (2013) Touching base with PARPs: moonlighting in the repair of UV lesions and double‐strand breaks. Trends Biochem Sci 38, 321–330 23562323 10.1016/j.tibs.2013.03.002

[15] Pines A, Vrouwe MG, Marteijn JA, Typas D, Luijsterburg MS, Cansoy M, Hensbergen P, Deelder A, de Groot A, Matsumoto S*et al* (2012) PARP1 promotes nucleotide excision repair through DDB2 stabilization and recruitment of ALC1. J Cell Biol 199, 235–249 23045548 10.1083/jcb.201112132 PMC3471223

[16] Luijsterburg MS, Lindh M, Acs K, Vrouwe MG, Pines A, van Attikum H, Mullenders LH & Dantuma NP (2012) DDB2 promotes chromatin decondensation at UV‐induced DNA damage. J Cell Biol 197, 267–281 22492724 10.1083/jcb.201106074 PMC3328393

[17] King BS, Cooper KL, Liu KJ & Hudson LG (2012) Poly(ADP‐ribose) contributes to an association between poly(ADP‐ribose) polymerase‐1 and xeroderma pigmentosum complementation group A in nucleotide excision repair. J Biol Chem 287, 39824–39833 23038248 10.1074/jbc.M112.393504 PMC3501068

[18] Kamileri I, Karakasilioti I & Garinis GA (2012) Nucleotide excision repair: new tricks with old bricks. Trends Genet 28, 566–573 22824526 10.1016/j.tig.2012.06.004

[19] Epstein JH & Cleaver JE (1992) 3‐Aminobenzamide can act as a cocarcinogen for ultraviolet light‐induced carcinogenesis in mouse skin. Cancer Res 52, 4053–4054 1617682

[20] Wakasugi M, Kasashima H, Fukase Y, Imura M, Imai R, Yamada S, Cleaver JE & Matsunaga T (2009) Physical and functional interaction between DDB and XPA in nucleotide excision repair. Nucleic Acids Res 37, 516–525 19056823 10.1093/nar/gkn964 PMC2632899

[21] Camenisch U & Nageli H (2008) XPA gene, its product and biological roles. Adv Exp Med Biol 637, 28–38 19181108 10.1007/978-0-387-09599-8_4

[22] Pleschke JM, Kleczkowska HE, Strohm M & Althaus FR (2000) Poly(ADP‐ribose) binds to specific domains in DNA damage checkpoint proteins. J Biol Chem 275, 40974–40980 11016934 10.1074/jbc.M006520200

[23] Fahrer J, Kranaster R, Altmeyer M, Marx A & Bürkle A (2007) Quantitative analysis of the binding affinity of poly(ADP‐ribose) to specific binding proteins as a function of chain length. Nucleic Acids Res 35, e143 17991682 10.1093/nar/gkm944 PMC2175335

[24] Popp O, Veith S, Fahrer J, Bohr VA, Bürkle A & Mangerich A (2013) Site‐specific noncovalent interaction of the biopolymer poly(ADP‐ribose) with the Werner syndrome protein regulates protein functions. ACS Chem Biol 8, 179–188 23082994 10.1021/cb300363g PMC3549037

[25] Antolin AA, Jalencas X, Yelamos J & Mestres J (2012) Identification of pim kinases as novel targets for PJ34 with confounding effects in PARP biology. ACS Chem Biol 7, 1962–1967 23025350 10.1021/cb300317y

[26] Madison DL, Stauffer D & Lundblad JR (2011) The PARP inhibitor PJ34 causes a PARP1‐independent, p21 dependent mitotic arrest. DNA Repair (Amst), 10, 1003–13 21840268 10.1016/j.dnarep.2011.07.006 PMC3185120

[27] Burger K, Matt K, Kieser N, Gebhard D & Bergemann J (2010) A modified fluorimetric host cell reactivation assay to determine the repair capacity of primary keratinocytes, melanocytes and fibroblasts. BMC Biotechnol 10, 46 20569452 10.1186/1472-6750-10-46 PMC2900224

[28] Malanga M, Pleschke JM, Kleczkowska HE & Althaus FR (1998) Poly(ADP‐ribose) binds to specific domains of p53 and alters its DNA binding functions. J Biol Chem 273, 11839–11843 9565608 10.1074/jbc.273.19.11839

[29] Trautlein D, Deibler M, Leitenstorfer A & Ferrando‐May E (2010) Specific local induction of DNA strand breaks by infrared multi‐photon absorption. Nucleic Acids Res 38, e14 19906733 10.1093/nar/gkp932 PMC2817483

[30] Trautlein D, Adler F, Moutzouris K, Jeromin A, Leitenstorfer A & Ferrando‐May E (2008) Highly versatile confocal microscopy system based on a tunable femtosecond Er:fiber source. J Biophotonics 1, 53–61 19343635 10.1002/jbio.200710019

[31] Mortusewicz O, Ame JC, Schreiber V & Leonhardt H (2007) Feedback‐regulated poly(ADP‐ribosyl)ation by PARP‐1 is required for rapid response to DNA damage in living cells. Nucleic Acids Res 35, 7665–7675 17982172 10.1093/nar/gkm933 PMC2190722

[32] Campalans A, Kortulewski T, Amouroux R, Menoni H, Vermeulen W & Radicella JP (2013) Distinct spatiotemporal patterns and PARP dependence of XRCC1 recruitment to single‐strand break and base excision repair. Nucleic Acids Res 41, 3115–3129 23355608 10.1093/nar/gkt025 PMC3597691

[33] Gibson BA & Kraus WL (2012) New insights into the molecular and cellular functions of poly(ADP‐ribose) and PARPs. Nat Rev Mol Cell Biol 13, 411–424 22713970 10.1038/nrm3376

[34] Gagne JP, Isabelle M, Lo KS, Bourassa S, Hendzel MJ, Dawson VL, Dawson TM & Poirier GG (2008) Proteome‐wide identification of poly(ADP‐ribose) binding proteins and poly(ADP‐ribose)‐associated protein complexes. Nucleic Acids Res 36, 6959–6976 18981049 10.1093/nar/gkn771 PMC2602769

[35] Buchko GW, Ni S, Thrall BD & Kennedy MA (1998) Structural features of the minimal DNA binding domain (M98‐F219) of human nucleotide excision repair protein XPA. Nucleic Acids Res 26, 2779–2788 9592168 10.1093/nar/26.11.2779 PMC147584

[36] Ikegami T, Kuraoka I, Saijo M, Kodo N, Kyogoku Y, Morikawa K, Tanaka K & Shirakawa M (1998) Solution structure of the DNA‐ and RPA‐binding domain of the human repair factor XPA. Nat Struct Biol 5, 701–706 9699634 10.1038/1400

[37] Nocentini S, Coin F, Saijo M, Tanaka K & Egly J‐M (1997) DNA damage recognition by XPA protein promotes efficient recruitment of transcription factor II H. J Biol Chem 272, 22991–22994 9287294 10.1074/jbc.272.37.22991

[38] Park C‐H, Mu D, Reardon JT & Sancar A (1995) The general transcription‐repair factor TFIIH is recruited to the excision repair complex by the XPA protein independent of the TFIIE transcription factor. J Biol Chem 270, 4896–4902 7876263 10.1074/jbc.270.9.4896

[39] Gagne JP, Pic E, Isabelle M, Krietsch J, Ethier C, Paquet E, Kelly I, Boutin M, Moon KM, Foster LJ*et al* (2012) Quantitative proteomics profiling of the poly(ADP‐ribose)‐related response to genotoxic stress. Nucleic Acids Res 40, 7788–7805 22669911 10.1093/nar/gks486 PMC3439892

[40] Zhang Y, Wang J, Ding M & Yu Y (2013) Site‐specific characterization of the Asp‐ and Glu‐ADP‐ribosylated proteome. Nat Methods 10, 981–984 23955771 10.1038/nmeth.2603

[41] Weaver AN & Yang ES (2013) Beyond DNA repair: additional functions of PARP‐1 in cancer. Front Oncol 3, 290 24350055 10.3389/fonc.2013.00290 PMC3841914

[42] Rajamohan SB, Pillai VB, Gupta M, Sundaresan NR, Birukov KG, Samant S, Hottiger MO & Gupta MP (2009) SIRT1 promotes cell survival under stress by deacetylation‐dependent deactivation of poly(ADP‐ribose) polymerase 1. Mol Cell Biol 29, 4116–4129 19470756 10.1128/MCB.00121-09 PMC2715814

[43] Bai P, Canto C, Oudart H, Brunyanszki A, Cen Y, Thomas C, Yamamoto H, Huber A, Kiss B, Houtkooper RH*et al* (2011) PARP‐1 inhibition increases mitochondrial metabolism through SIRT1 activation. Cell Metab 13, 461–468 21459330 10.1016/j.cmet.2011.03.004 PMC3086520

[44] Fan W & Luo J (2010) SIRT1 regulates UV‐induced DNA repair through deacetylating XPA. Mol Cell 39, 247–258 20670893 10.1016/j.molcel.2010.07.006

[45] Porter PC, Mellon I & States JC (2005) XP‐A cells complemented with Arg228Gln and Val234Leu polymorphic XPA alleles repair BPDE‐induced DNA damage better than cells complemented with the wild type allele. DNA Repair (Amst) 4, 341–349 15661657 10.1016/j.dnarep.2004.10.007

[46] Tao GH, Yang LQ, Gong CM, Huang HY, Liu JD, Liu JJ, Yuan JH, Chen W & Zhuang ZX (2009) Effect of PARP‐1 deficiency on DNA damage and repair in human bronchial epithelial cells exposed to Benzo(a)pyrene. Mol Biol Rep 36, 2413–2422 19247804 10.1007/s11033-009-9472-z

[47] Mellon I, Hock T, Reid R, Porter PC & States JC (2002) Polymorphisms in the human xeroderma pigmentosum group A gene and their impact on cell survival and nucleotide excision repair. DNA Repair (Amst) 1, 531–546 12509227 10.1016/s1568-7864(02)00053-8

[48] Noren Hooten N, Kompaniez K, Barnes J, Lohani A & Evans MK (2011) Poly(ADP‐ribose) polymerase 1 (PARP‐1) binds to 8‐oxoguanine‐DNA glycosylase (OGG1). J Biol Chem 286, 44679–44690 22057269 10.1074/jbc.M111.255869 PMC3247967

[49] Noren Hooten N, Fitzpatrick M, Kompaniez K, Jacob KD, Moore BR, Nagle J, Barnes J, Lohani A & Evans MK (2012) Coordination of DNA repair by NEIL1 and PARP‐1: a possible link to aging. Aging (Albany NY) 4, 674–685 23104860 10.18632/aging.100492 PMC3517938

[50] Beneke S, Scherr AL, Ponath V, Popp O & Bürkle A (2010) Enzyme characteristics of recombinant poly(ADP‐ribose) polymerases‐1 of rat and human origin mirror the correlation between cellular poly(ADP‐ribosyl)ation capacity and species‐specific life span. Mech Ageing Dev 131, 366–369 20399804 10.1016/j.mad.2010.04.003

[51] Kiehlbauch CC, Aboul‐Ela N, Jacobson EL, Ringer DP & Jacobson MK (1993) High resolution fractionation and characterization of ADP‐ribose polymers. Anal Biochem 208, 26–34 8434792 10.1006/abio.1993.1004

[52] Missura M, Buterin T, Hindges R, Hubscher U, Kasparkova J, Brabec V & Naegeli H (2001) Double‐check probing of DNA bending and unwinding by XPA‐RPA: an architectural function in DNA repair. EMBO J 20, 3554–3564 11432842 10.1093/emboj/20.13.3554 PMC125508

[53] Burger K, Kieser N, Gallinat S, Mielke H, Knott S & Bergemann J (2007) The influence of folic acid depletion on the nucleotide excision repair capacity of human dermal fibroblasts measured by a modified host cell reactivation assay. BioFactors 31, 181–190 18997281 10.1002/biof.5520310305

